# Initial forest age distribution may generate computational sinks or sources of carbon: A generic approach to test assumptions underlying the EU LULUCF forest reference levels

**DOI:** 10.1186/s13021-021-00177-4

**Published:** 2021-05-04

**Authors:** Jari Vauhkonen, Antti Mutanen, Tuula Packalen, Antti Asikainen

**Affiliations:** 1 Department of Forest Sciences, University of Helsinki, Latokartanonkaari 7, P.O. Box 27, 00014 Helsinki, Finland; 2 Natural Resources Institute Finland (Luke), Yliopistokatu 6 B, 80100 Joensuu, Finland; 3 Ministry of Agriculture and Forestry, Natural Resources Department, P.O. Box 30, 00023 Government, Finland; 4 Natural Resources Institute Finland (Luke), Yliopistokatu 6 B, 80101 Joensuu, Finland

**Keywords:** Land use, Land use change and forest, Biomass available for wood supply, Harvest fraction of management, Harvesting intensity, European Forestry Dynamics Model (EFDM), Chapman‐richards function, Growth and yield model, Simulation, Projection, Modelling

## Abstract

**Background:**

The current EU LULUCF regulation calls for member state-specific Forest Reference Levels (FRLs) for benchmark in the accounting of greenhouse gas emissions and removals of managed forest land during the compliance period (2021–2030). According to the technical guidance on developing and reporting the FRLs, it could be actualized by projecting a ratio of harvested to total available biomass. We tested how the initial age distribution may affect the aforementioned ratio by simulating the continuation of forest management based on several descriptive shapes of forest age class distribution.

**Results:**

Our simulations suggest that when the FRLs are prepared by employing the harvest ratio and forest management is assumed strictly age dynamics driven, the shape of the initial forest age class distribution gives rise to computational sinks or sources of carbon in managed forest land. Harvests projected according to the ratio corresponded those resulting from the age dynamics only in the case of uniform age distribution.

**Conclusions:**

The result calls for a better consideration of variation in initial states between countries when determining the future LULUCF regulation. Our exercise demonstrates how generic simulations in a standardized modeling framework could help in *ex-ante* impact assessment of proposed changes to the LULUCF regulation.

**Supplementary Information:**

The online version contains supplementary material available at 10.1186/s13021-021-00177-4.

## Background

The EU LULUCF regulation [1] provides the inclusion of greenhouse gas (GHG) emissions and removals from land use, land use change, and forestry into the EU’s 2030 climate and energy framework. Key elements in the regulation are the commitment that the accounted GHG emissions from the LULUCF sector shall not exceed the accounted removals (i.e., the no debit rule), the rules on how emissions and removals are accounted for in different land accounting categories, and the flexibilities which the member states may utilize in order to meet the no debit rule. The legislative process of the regulation was complex and required about two years of intensive negotiations in the institutions of the EU. Since the adoption of the LULUCF regulation in May, 2018, the EU has started a process of revising its climate change and energy framework. The intensifying targets for mitigating the climate change challenge the member states to enhance and increase the sink capacity within the LULUCF sector. Accordingly, also the EU LULUCF regulation needs a revision as pointed out in a timely and profound summary [2].

The GHG accounting rules of the LULUCF regulation vary between land categories. For afforested land and deforested land, total emissions and total removals are accounted for during the compliance period (gross-net accounting). In other land categories, the sum of total emissions and removals during the compliance period is compared with a reference level (net-net accounting). Apart from the land category of managed forest land, the reference level is based on historical observations. In accounting the emissions and removals of managed forest land, member state-specific Forest Reference Levels (FRLs) are used. According to the regulation, the FRL “shall be based on the continuation of sustainable forest management practice, as documented in the period from 2000 to 2009 with regard to dynamic age-related forest characteristics in national forests, using the best available data”. In addition, the regulation states that the FRL “shall take account of the future impact of dynamic age-related forest characteristics in order not to unduly constrain forest management intensity as a core element of sustainable forest management practice, with the aim of maintaining or strengthening long-term carbon sinks”.

Several studies have considered the effects of the continuation of forest management and the related computations from different points of view. Following the legislative proposal of the regulation, Grassi et al. [3] proposed a methodological approach, which is in line with the regulation text, and assessed the FRLs for 26 EU Member States based on this approach implemented using the Carbon Budget Model (CBM). Nabuurs et al. [4], upon an analysis of harvesting possibilities based on the EFISCEN model, suggested that the 26 countries together can increase harvests complying the continuation of management practices criteria without creating debits. Forsell et al. [5] examined the role of different computational assumptions, finding technicalities such as the starting year for the projection, stratification criteria for the area of managed forest land, and simulated timing of individual management activities to have less impact compared to assumptions on climate change or allocation of management practices. For their analyses, Forsell et al. [5] coupled forest dynamics and GHG modelling based on the G4M and WoodCarbonMonitor frameworks.

Apart from studies focused on the FRLs in the EU-LULUCF context, more critical views on applying the continuation of forest management in future projections have been expressed. Vauhkonen and Packalen [6] suggested that the continuation of past management could lead to inefficient decisions regarding future carbon stocks and harvesting possibilities. Their conclusion was based on adding climate- or management-induced growth improvements (and no opposite effects) on projections of forest age dynamics using the European Forestry Dynamics Model (EFDM). Then again, Nabuurs et al. [4] noted that when combined with a progressing age class development over time, the continuation of forest management could lead to approaching or exceeding maximum sustained harvest amounts. As an alternative to constant management intensity derived from past data, Nabuurs et al. [4] analyzed harvests cut off based on a removal/increment ratio that might not comply with regulation [5].

We stress some additional issues that need to be considered when preparing, interpreting, and evaluating the FRL projections. First, the technical guidance on developing and reporting the FRLs promotes an idea that the continuation of forest management could be condensed to a harvesting intensity, or Harvest Fraction of Management (HFM) [7] that is computed from the Reference Period (RP; years 2000–2009) and applied to the projected age class distribution of the Compliance Period (CP; 2021–2030). This recommendation as well as all the results listed in the above paragraphs might not be independent of the initial age class distribution of the EU member state(s) in question.

Second, challenges may be related to understanding these definitions and projecting forest resources, harvests, and FRLs accordingly. The EU member states differ in terms of forest characteristics, forestry practices, quality of forest inventory data, and modeling experience [5] that results to applying various methods and reduced comparability of the FRL proposals [2]. According to text above, many different projection models were used for studying the fundamentals of the FRL principles, even though such assessments would benefit from harmonized definitions and standardized modeling (cf., [8]). According to an assessment of the FRLs submitted by altogether 28 member states [9], the CBM model was attributed as the forest projection approach of three member states, while the remainder used disparate models and altogether 8 approaches categorized as “*ad hoc* FRL models” (Table 3 in [9]).

We would like to add to the discussion on the revision needs of the LULUCF regulation [2] by showing an example on how the above challenges in the current specification of the FRLs may affect the computational outcomes. We aim to demonstrate (*i*) how the initial age distribution may affect the harvesting intensity underlying the FRLs and (*ii*) how reproducible code in a standardized modeling framework could benefit similar analyses and discussion on potential defects of the FRL principles, thus helping to revise these principles in the future.

The specific idea and overview of the study are described as follows. To treat the member states in a fair manner, it is reasonable to require that the FRL principles are unspecific to an age distribution of any member state or even that of the member states on average. By generating hypothetical forest areas of (arbitrary) 10,000 km^2^, we gained access to data not focused or limited to actual forest structure of any specific EU member state that can be used to generically test and communicate the assumptions and principles underlying the FRLs. We assumed the simulated forests to have a single species in a single stratum with the initial age distribution as the only distinctive feature. The forest development was assumed to be solely age-class driven without assumed objectives to optimize the rotation or regulate a forest, i.e. convert forest with an unbalanced age class distribution into one with equal area in each age class. As the age distributions, we sampled random values from six descriptive distribution shapes, which were additionally initialized to different years by means of back- and forecasting, to yield altogether 12 different input distributions for the simulations. We used the EFDM to project the FRL-compliant, dynamic age-related forest characteristics [7] by moving forest areas from an age class to another over time. Subsequently, the only management action was to harvest the oldest age class in a time step. We recorded the harvests and growing stock resulting from projecting this type of continuation of forest management to each age class distribution for 65 years. We compare this development to a HFM projected from the RP to the future, which is proposed to sufficiently proxy for the continuation of forest management in the current FRL guidance [7].

## Results

**Fig. 1:**
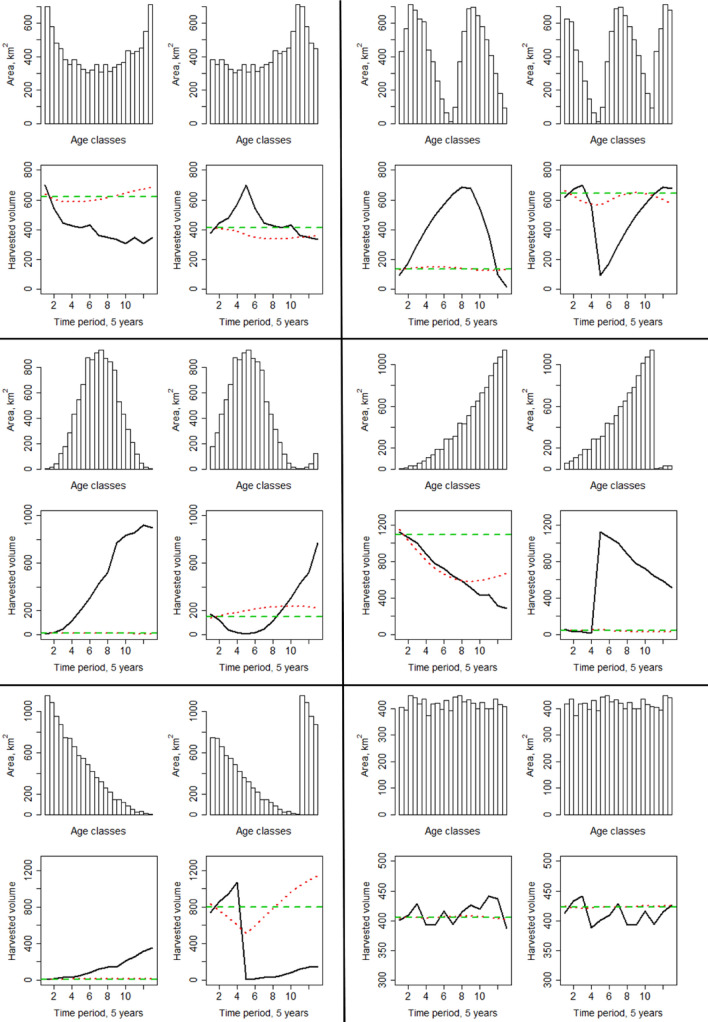
The effect of the descriptive shape of the age class distribution to the harvesting intensity obtained by the Harvest Fraction of Management (HFM). The upmost row of each panel illustrates the different distributions of age classes in year 2000: left-hand shows the distribution obtained directly for year 2000 and right-hand one obtained for 2020 and back-cast to 2000. The bottom row shows the harvested volume, when it is obtained either as the volume of the oldest age class of each time step (black line), the ratio of harvested to total volume during the RP (HFM_RP_; green line), or HFM_RP_ multiplied by the total volume of each time step (red line). The Reference Period (RP) and Compliance Period (CP) took place in the periods 1–2 and 5–6, respectively. The six panels correspond to, in clockwise order from the upper left corner, projections of the bimodal (*bimod1* and *bimod2*), reverse-J shaped (*revj*), uniform (*unif*), J-shaped (*skwj*), and normal (*norm*) distributions

In our simulations (see Methods), forest management contributes to a FRL as a computational source of carbon, when the harvests realized in the CP exceed those obtained by projecting the ratio of harvested to total available biomass of the RP, and a computational sink otherwise. Among the simulated age class distributions, 5 were computational sources and 7 sinks of carbon during the CP (yet, the result is clearly a tie between the forecast and backcast versions of all other distributions except the uniform distribution, for which it depends on the small probabilistic variation around the uniformity). Figure 1 shows results based on different descriptive shapes of the age class distributions.

According to Fig. 1, the conformity of harvestable age classes between the RP and CP is greatly affected by how much the HFM diverged from the realized harvests due to the age class dynamics. Among different age class distributions, the projected HFM corresponded with the harvesting intensity resulting from the age dynamics only in the case of the uniformly distributed forest (Fig. 1, lower right panel). Even if the projected HFM somewhat allowed reacting to dynamics due the underlying age distribution, this reaction most often differed in timing and magnitude from that based on the age class dynamics. A comparison between the results obtained as either fore- or back-casting indicated that possible peaks of the age class distribution caused the forest to be either a source or sink depending on the timing of the peak between the RP and CP (Fig. 1).

**Fig. 2:**
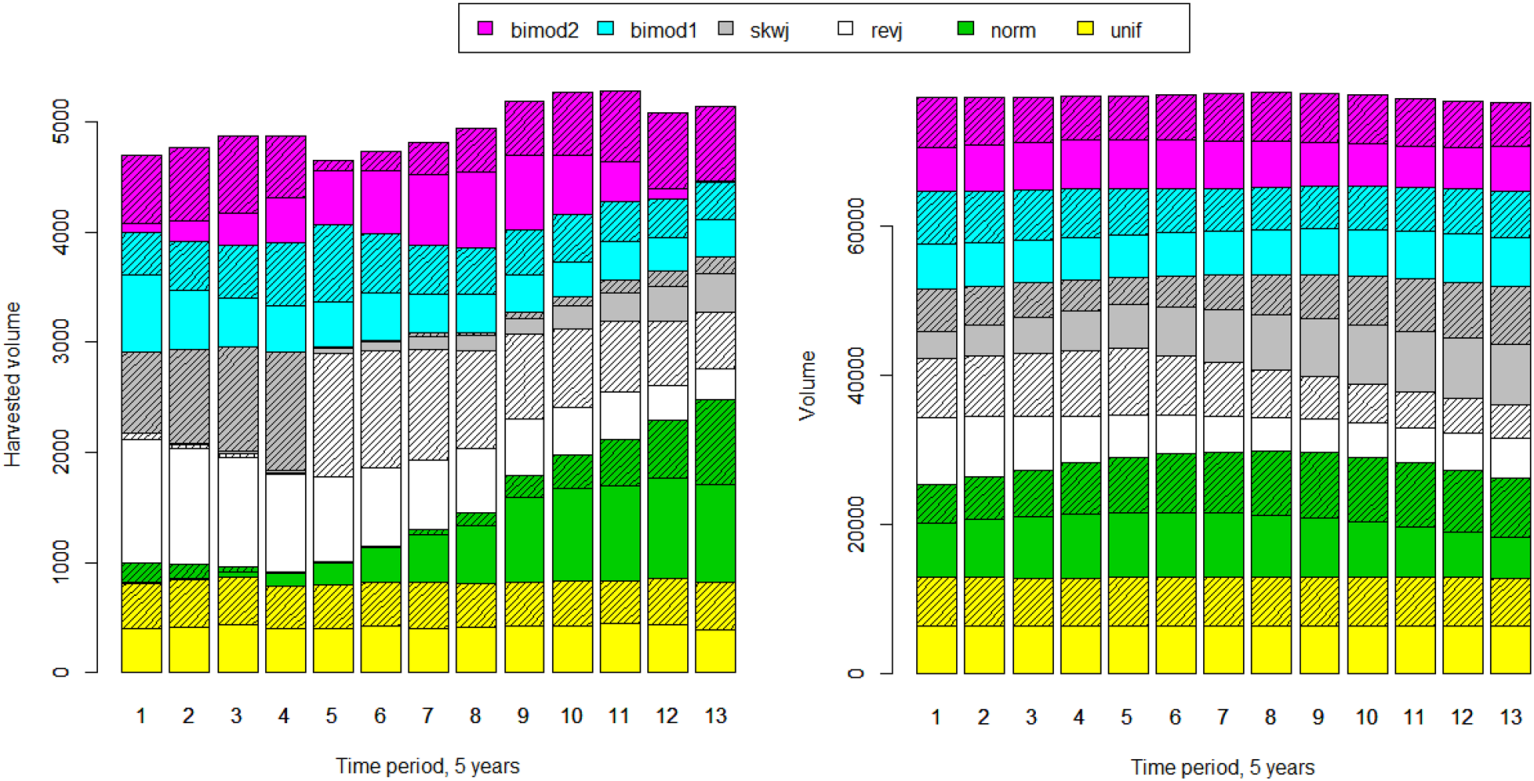
The total volume harvested (left) and growing stock (right), when harvesting the oldest class in a time step for altogether 13 five-year periods. The Reference Period (RP) and Compliance Period (CP) took place in the periods 1–2 and 5–6, respectively. The filled and dashed bars correspond to forecast and backcast, respectively, projections of the bimodal (*bimod1* and *bimod2*), J-shaped (*skwj*), reverse-J shaped (*revj*), normal (*norm*), and uniform (*unif*) distributions. The values of the y-axis are arbitrary total volumes that depend on the age-volume relationship (see "Methods")

Figure 2 shows the development of the harvested and growing stock volume of the different age class distributions altogether, i.e., at an area of (12 distributions × 10,000 km^2^ =) 120,000 km^2^. The distribution of the forest area to the age classes (in particular, the area in the oldest age class) determined the management need and led to abrupt changes in harvested volume especially for the skewed distributions (Fig. 2). Even if these changes due to implementing management according to the underlying age class dynamics were reflected on the harvested volume of the entire area, the growing stock volume remained steady over the time horizon as well as between the simulation steps

## Discussion

In above, both the realized and projected continuation of the forest management practice and intensity were simulated strictly according to age dynamics, i.e., harvesting the oldest age class in a time step. In other words, the only simulated management action was final felling, whereas a portion of harvest removals of many countries come from various types of thinnings. The simulation can thus be claimed simplistic; however, it involves the key aspects of the FRL, i.e., continuation of forest management in the RP based on a regime that is strictly determined by the age distribution during the entire simulation. From the technical point of view, thinnings can be easily added to the code producing the transition and activity matrices for the simulations. However, this addition requires determining which proportion of area in each age class is thinned and how the thinning affects (how and where the area in the age and volume classes transits due to the thinning), i.e. many more assumptions that do not directly depend from or affect the age class distribution. Because of the openly available implementation, any interested reader can change assumptions related to input data, management actions or evaluation criteria to, for instance, evaluate the correctness or correspondence of this example with real-world data.

The FRLs aim at recording the carbon impact from a deviation of forest management relative to the RP, whereas the related policy aims at carbon impacts at the level of the EU as a whole. Therefore, it was interesting to examine the joint regulating potential of the distributions generated (Fig. 2). In the following text, it is mainly explored from the computational-technical point of view of the FRL, but we also note the related political dimension of the regulation (see below). Assume that the individual age class distributions reflected a selection of EU member states that become regulated according to the LULUCF FRLs. Then the harvest amounts resulting from age-class driven dynamics become either computational sinks or sources of carbon according to Fig. 1. The approximately equal amount of sinks and sources could possibly generate trading between member states to meet their obligations (e.g., compare the opposite magnitudes of harvests of the distributions *revj* and *skwj* in Fig. 2). Nevertheless, both positive and negative deviations to the total harvest volumes relative to the FRLs are allowable under the LULUCF regulation. When the changes in the harvest volumes reflect the underlying changes in the age structure, these state-specific deviations might have no positive or negative effect to the total carbon storage of the entire area (Fig. 2).

The message of the previous paragraph should be explored from another perspective. According to Fig. 2, if the projected continuation of forest management perfectly captured that realized, it would suit well for a benchmark for human-added efforts to enhance the carbon sinks at the global level (‘global’ referring here to the simulated age distributions altogether). However, when the global level consists of forests at the different state of development, both the growing stock and harvesting possibilities are divided unequally according to the forest age distributions. According to our results, projecting a ratio of harvested to total available biomass, which is proposed as the proxy of the continuation of forest management practice in the guidance to develop the FRLs [7], may not be flexible enough to adjust according to natural age class dynamics (Fig. 1). A possible consequence is that the FRLs computed for the CP may treat member states with different initial forest age structure unequally. In our simulations, this is a joint effect of varying initial forest age classes, the FRL principle to continue the past management, and the simplified HFM metric proposed in the technical guidance for projecting the continuation of forest management.

According to our results, continuing the forest management of the RP may not be a proper choice for the projections of the CP because different management actions are needed due to differences in the age class distribution between RP and CP. In fact, the EU LULUCF regulation also recognizes the future aspect and the dynamics of forests’ age-related characteristics while stating that the FRLs should not unduly constrain the intensity of forest management when maintaining or strengthening the carbon sinks. Depending on the age structure, this statement may not realize with a strict continuation of the forest management practice from the RP. There are further inconsistencies in the guidance on what can and what cannot be included in the projections of the FRLs. The projections are allowed to assume future climatic conditions in the CP according to country-specific climate projections, whereas “the assumed future impact of policies and markets are not to be included” [7]. However, future forest resources and the degree of them that is available for different uses such as wood production or carbon sequestration evolve according to both the markets and climate. In turn, both affect future management regimes and even ownership structure determining the development of forest resources via complex interactions. Therefore, excessive emphases on accurate reproduction of past management practices (hereafter, referred to as the backward-looking nature of the current FRLs) can be in contrast to implementing sustainable forest management [4] or enhancing future carbon stocks [6]. This aspect becomes problematic also if an ability to correctly produce the historical development of the forest resources (in the RP or before the CP) becomes a key success criterion for the FRL proposals.

It is estimated that the EU carbon removals will need to nearly double from their current level to up to 500 Mt CO2eq./a by 2050 to be in line with aspirations for a climate-neutral EU [10]. However, the carbon removal capacity of EU’s forests is estimated to decrease due to ageing, harvest and large scale forest disturbances such as fires, insect outbreaks and extreme weather conditions [11]. Further studies should consider FRLs from the perspectives of effects of (1) natural disturbances and (2) markets and forestry policies that differ between member states except for the regulating needs. On the latter point, the reader is directed to a recent discussion on applying FRLs for GHG accounting in competitive global markets (see [12] and references therein). These factors should be incorporated into modelling efforts when redesigning the EU’s LULUCF regulation to avoid its possible adverse effects to practice sustainable forest management.

The above text suggests that it could be beneficial to re-consider the backward-looking nature of the principles and definitions of the FRLs. Obviously, challenges are also related to implementing any forward-looking approach. Accounting for the aforementioned factors is more climate–market modeling than forestry modeling. For scenario analyses, the multiple unknown factors that affect the future outcomes should be included in the assumptions of the projections [13–15]. For instance, Nabuurs et al. [16] suggest 50–60 years as a maximum feasible time span for the projections. However, this conclusion is based on reproducing historical development of 1923–1963 and qualitatively analyzing scenarios run until 2050 but parameterized by forest inventory data until 1990’s. The maximum feasible time span under more recent uncertainties related to markets (e.g. covid-19 market disturbance), climate and management may be considerably shorter, or it should at least be re-evaluated. The current FRLs are prepared for a time span of 30 years from the beginning of RP to the end of CP, but if the maximum feasible time span at which the aforementioned factors hold turns out to be markedly shorter, it should be reflected to the FRL principles, for example.

For our exercise, the EFDM was selected as the modeling tool mainly for its flexible open-source implementation. However, according to a test that involved NFIs of 20 European countries [8], the EFDM is a viable option for international modelling exercises. Even if not demonstrated specifically for the FRL computation, projections that consistently up-/downscale between country and European levels were prepared based on harmonized definitions, assumptions, and modelling methodology, while maintaining country-specific forestry practices [8]. On the other hand, by varying these assumptions to account for the related uncertainty [6, 17], it is possible to analyze an interval of possible future outcomes as basis for forward-looking FRLs. Developing FRL guidance based on open-source modeling environments would allow utilizing pseudo-code and metadata templates [18] for conveying the modeling instructions and assumptions that would further promote the transparency of the reporting.

It should be emphasized that the FRLs are not binding targets but benchmark values used in and created for the LULUCF accounting framework and as such do not directly affect the forest management in the member states. Obviously, both the positive and negative deviations from the FRL are allowable, but when a deviation results in accounted emissions of the managed forest land category, it needs to be compensated. Compensation is automatic within the LULUCF sector between the land use categories having calculated positive net removals. Compensation is also possible via Effort Sharing sector or via transacting surplus net removals of the LULUCF sector between the other member states, and in addition, via a country-specific managed forest land flexibility that is provided to dampen the effects due to the technicalities related to the FRLs (see article 12 and 13 of [1] with the related Annex VII). Nevertheless, our study shows that the FRLs based strictly on age class driven forest management practices and the HFM metric may lead to unequal results between member states and hamper the EU-level target of maintaining and enhancing of carbon sinks. Thus, based on a purely technical evaluation, some of these potentially adverse consequences could possibly have been avoided by different formulations of the FRL guidelines.

## Conclusions

In our computational exercise, the initial forest age class distribution greatly affected the Harvest Fraction of Management [7] proxy proposed for developing FRLs under the EU LULUCF regulation. Among the different descriptive shapes of the age distribution simulated, only the uniformly distributed forest yielded a harvesting intensity, the projection of which approximately corresponded with the realized continuation of the forest management. Otherwise, the asymmetry of the distribution with respect to the Reference and Compliance Periods gave rise to computational sinks or sources of carbon, while the Harvest Fractions of Management of the aforementioned periods were not flexible enough to adjust to the age class dynamics.

The deviations from the uniformity in the real-world, country-specific forest age class distributions may specifically be drivers of forest policy aiming to modify the distribution, which can further partially affect the difficulty to compare the FRL proposals [2]. The temporal differences in age class dynamics and variation due to climate, markets, and management between the reference and compliance periods hinder the possibilities to validate projections against past inventory data. The possible revision of the EU-LULUCF regulation [2] should consider possibilities to define FRLs to be less dependent on the initial forest age class distribution.

By simulating forest age dynamics upon generated age class distributions, we gained access to data on both realized and projected management that is not limited to an actual age distribution of EU member state(s). We demonstrated how reproducible code in a standardized, open-source modeling framework could benefit similar analyses, discussion, and potential revision processes.

## Methods

The aim of this study was to simulate the continuation of forest management according to both the age dynamics of generic age class distributions and a FRL-compliant harvesting intensity derived from the distributions. We considered hypothetical forest areas of 10,000 km^2^ distributed to 24 age classes of five years. Six different descriptive shapes of this distribution were generated by drawing random samples from the Beta distribution with shape parameters selected to produce the uniform, normal, J-shaped, reverse-J shaped, and two different bimodal distributions.

The age class distributions were projected forward according to transition matrices that implement three steps of a standard text book method: in each simulation step, (1) harvest the oldest age class; (2) move the total harvested area into the youngest age class; (3) move the uncut area up to the next age class. The continuation of this type of forest management will revert the distribution back to the initial in one rotation (24 age classes × 5 years = altogether 120 years). The transition matrices inherently included two dimensions (age and volume), but only the transitions of age classes were meaningfully implemented and the volume was obtained as a function of age (see below). The volume harvested in a given period consequently depended on the area of the oldest age class that varied as per distribution generated.

The growing stock volume (*V*) was assumed to develop as a function of age according to a Chapman-Richards equation [e.g., 19], *V* = *vmax* × (1 + *e*^*rate* × t^)^*shape*^, where parameters *vmax*, *rate* and *shape* received values 1.0, -0.05, and 5.0, respectively, and *t* was the middle point of an age class in years. The parameter values were selected to produce a generic, sigmoid-form transformation from age to volume, yielding proportions of juvenile forest with an accelerating rate of growth; matured, vigorous forest with a constant rate of growth; and senescent forest with a decelerating rate of growth. The parameter *vmax* corresponds to the upper asymptote of the curve, i.e., the maximum value the volume can take as a function of age approached to one. For example, having all of the 10,000 km^2^ of forest in the oldest class would have yielded an arbitrary total volume of 9,877. We additionally ran tests with *shape* = 1 and *shape* = 10 as well as the yield function by Fridh and Nilsson [20] to convince ourselves that except for the total growing stock value, the effects discussed here were caused by the descriptive shape of the age class distribution.

The generated data were assumed to represent the state of the forest both in year 2000 and 2020, corresponding to the beginning of the RP and CP, respectively. In the latter case, the distribution was backward projected (or backcast, cf. [21]) with inverse transition matrices for four time steps so that the projections always started from year 2000. Accordingly, we obtained (with 6 distribution shapes × 2 starting years) altogether 12 simulations of 13 five-year periods (assumed to correspond to years 2000–2065), of which the RP and CP took place in the periods 1–2 and 5–6, respectively. The projections described above can be replicated by downloading the EFDM, v. 2.0, from https://github.com/ec-jrc/efdm and running it in the R statistical computing environment [22, 23] using the generated distributions as input. Code that generates the distributions and prepares input files for the projections is provided as additional material (see Additional file 1). By default, the input files are for the forecasting option, whereas the backcasting needs to be set up by removing the comment signs from rows 113–131 of the code before running it.

We used the EFDM outputs to compute the HFM [7] to project the harvests from the RP to the CP in compliance with the FRL. Specifically, we implemented “Alternative 2” [7, p. 60], which relates the total harvested biomass (H) to the total biomass available (TBA) instead of more specific “biomass available for wood supply” [3]. Accordingly, the ratio HFM_RP_ = H_RP_ / TBA_RP_ can be used to determine the allowable future harvest in the CP as HFM_RP_ × TBA_CP_. Because biomass is merely volume × expansion factor, we omitted this conversion and demonstrate our results in terms of the growing stock volume.

## Supplementary Information

**Additional file 1.** R-code used to generate the forest age class distributions and prepare the EFDM input data.

## Data Availability

The study is based on simulated data that can be regenerated by running the code presented as Additional file 1.
